# Tissue factor pathway inhibitor for prediction of placenta-mediated adverse pregnancy outcomes in high-risk women: AngioPred study

**DOI:** 10.1371/journal.pone.0173596

**Published:** 2017-03-22

**Authors:** Aurélie Di Bartolomeo, Céline Chauleur, Jean-Christophe Gris, Céline Chapelle, Edouard Noblot, Silvy Laporte, Tiphaine Raia-Barjat

**Affiliations:** 1 Department of Gynaecology and Obstetrics, University Hospital, Saint Etienne, France; 2 INSERM UMR 1059, Saint Etienne University Jean Monnet, Saint Etienne, France; 3 Laboratory of Haematology, University Hospital, Nîmes, France; 4 Research Unit EA2992, Montpellier University, Montpellier, France; 5 Clinical Pharmacology Unit, University Hospital, Saint Etienne, France; Stellenbosch University, SOUTH AFRICA

## Abstract

**Objective:**

The study aimed to evaluate if the rate of tissue factor pathway inhibitor during pregnancy and following delivery could be a predictive factor for placenta-mediated adverse pregnancy outcomes in high-risk women.

**Methods:**

This was a prospective multicentre cohort study of 200 patients at a high risk of occurrence or recurrence of placenta-mediated adverse pregnancy outcomes conducted between June 2008 and October 2010. Measurements of tissue factor pathway inhibitor resistance (normalized ratio) and tissue factor pathway inhibitor activity were performed for the last 72 patients at 20, 24, 28, 32, and 36 weeks of gestation and during the postpartum period.

**Results:**

Overall, 15 patients presented a placenta-mediated adverse pregnancy outcome. There was no difference in normalized tissue factor pathway inhibitor ratios between patients with and without placenta-mediated adverse pregnancy outcomes during pregnancy and in the post-partum period. Patients with placenta-mediated adverse pregnancy outcomes had tissue factor pathway inhibitor activity rates that were significantly higher than those in patients without at as early as 24 weeks of gestation. The same results were observed following delivery.

**Conclusion:**

Among high-risk women, the tissue factor pathway inhibitor activity of patients with gestational vascular complications is higher than that in other patients. Hence, these markers could augment a screening strategy that includes an analysis of angiogenic factors as well as clinical and ultrasound imaging with Doppler measurement of the uterine arteries.

## Introduction

Placenta-mediated adverse pregnancy outcomes (or placental vascular pathology (PVP)) represents a heterogeneous group of multisystem disorders that can be maternal [pre-eclampsia (PE); eclampsia; retroplacental hematoma; hemolysis; elevated liver enzymes; low platelets (HELLP) syndrome] or foetoplacental through placental hypoperfusion, leading to intrauterine growth retardation (IUGR) or oligohydramnios. PE complicates between 2% and 8% of pregnancies[1], making placenta-mediated adverse pregnancy outcomes fairly common. It is also responsible for many cases of foetomaternal morbidity and mortality, and a major contributor to premature births.[2] PE is associated with preterm delivery in 15–67% of cases, with IUGR in 10–25% of cases, and with neonatal mortality in 1%–2% of cases.[3] Strategies for predicting and preventing the occurrence of these potentially serious complications in patients and foetuses remain a major challenge. The introduction of closer monitoring, transfer to adapted level maternity, and antenatal steroids can significantly reduce maternal and foetal morbidity, which underscores the importance of early identification.[4] Once PE occurs, the only curative treatment is delivery, and although placenta-mediated adverse pregnancy outcomes risk factors are now well-known, there is still no validated screening strategy for predicting the occurrence of this pathology in high-risk patients.[5,6]

Pregnancy represents an acquired hypercoagulable state due to a higher concentration of coagulation factors, low levels of anticoagulant, and reduced fibrinolysis. For example, during pregnancy there is a physiological decrease in protein S and an increased expression of tissue factor (TF) [7]. TF plays a role in the extrinsic pathway of coagulation, because TF binds to FVII and increases its activity. The resulting TF/VIIa complex then activates factor X. This mechanism is regulated by TFPI (tissue factor pathway inhibitor) [8], a Kunitz-like protein produced mainly by endothelial cells. Factor Xa and the TF/VIIa complex are both inhibited by TFPI, 80% of which is found in the vascular endothelium, with the remaining 20% found in plasma (80% linked to lipoproteins and 20% free). Protein S enhances TFPI action [9,10], and studies have shown that, outside of pregnancy, modulation of TFPI rates is responsible for the increased risk of venous thromboembolism [11,12], particularly in women taking oral contraceptives [13,14]. However, only a few studies have been published that examine TFPI in the context of pregnancy, with contradictory results [15,16].

The main objective of our study was to evaluate the TFPI rate (TFPI resistance and TFPI activity) as a potential predictor of placenta-mediated adverse pregnancy outcomes in high-risk patients. The secondary objective was to evaluate TFPI evolution (TFPI resistance assay and TFPI activity assay) during 20–36 weeks of pregnancy and postpartum.

## Materials and methods

### Study population

The Angiopred study is a prospective multicentre cohort study of consecutive patients at a high risk of occurrence or recurrence of placenta-mediated adverse pregnancy outcomes, conducted between June 2008 and October 2010 at the Obstetrics and Gynaecology Department of Saint Etienne and Nîmes University Hospital and the Laboratory of Haematology at Nîmes University Hospital. Two hundred patients were included in this study. We performed analyzes of TFPI only on 72 patients who were included in Saint Etienne.

Inclusion criteria comprised a history of one or more of the following: (1) diabetes (dietary or insulin-dependent); (2) hypertension (previously treated before pregnancy or blood pressure > 140/90 at two readings prior to 20 weeks' gestation); (3) obesity (BMI ≥ 30) (4) maternal age ≤ 18 years or ≥ 38 years; (5) chronic kidney disease (proteinuria ≥ 300mg for 24 hours or creatinine ≥ 1.5 mg/dl prior to 20 weeks' gestation); (6) lupus; (7) antiphospholipid syndrome; (8) family history of cardiovascular disease or VTE; (9) biological thrombophilia without personal history of VTE or personal placenta-mediated adverse pregnancy outcomes; (10) a history of one or more episodes of PVP; and (11) a personal history of VTE. The exclusion criteria were as follows: (1) multiple pregnancies; (2) patients with a history of foetal death due to congenital malformations, Rh incompatibility, or infectious cause; (3) IUGR with a chromosomal, genetic, or infectious abnormality etiology; and (4) patients with placenta-mediated adverse pregnancy outcome at inclusion.

The protocol was approved by the Ethics Committee and Institutional Review Board of the University Hospital of Saint Etienne in March 2008 and all subjects provided written informed consent. This clinical investigation was performed according to the Helsinki declaration of 1975, as revised in 1996.

### Study design and evaluation criteria

At inclusion, patient demographic data were collected by interview, physical examination, and consultation of obstetrical medical records. Blood samples required by this study were taken alongside the conventional laboratory tests for monitoring pregnancy.

The outcome was the occurrence or recurrence of placenta-mediated adverse pregnancy outcomes in specific accordance with the following criteria: (1) foetal death at ≥ 20 weeks of gestation unrelated to foetal malformations, abnormal karyotype, or infectious disease; (2) PE defined according to the ISSHP (International Society for the Study of Hypertension in Pregnancy criteria);[17] (3) eclampsia defined as the presence of convulsions in a pre-eclamptic patient; (4) *abruptio placentae* defined by the presence of externalized haemorrhage and/or found on pathologic analysis of the placenta conducted in a context of preeclampsia, uterine contractions with no relaxation, or foetal distress; (5) HELLP syndrome; and (6) Small for Gestational Age defined by a birth weight ≤ the 10^th^ customised centile.

### Blood collection and laboratory methods

Blood samples were collected in tubes containing 0.129 mol L trisodium citrate, at the collection centre of the University Hospital of Saint Etienne and Nîmes, at 20, 24, 28, 32, and 36 weeks, and during the postpartum period, for a total of six samples per patient. Postpartum blood samples were performed 6 weeks after childbirth. After a single centrifugation (20 min; 2500 x g), 400 μL aliquots of plasma were collected and stored at −70°C until analysis. Each analysis was then performed blind to the other analyses. All samples from each patient were grouped in the same series of analyses. For the postpartum sample, two tubes were tested; one after the first centrifugation and one after the second, so there were ultimately seven samples per patient. Pooled normal (V) plasma from 34 healthy volunteers, excluding any women who were using oral contraceptives or who were pregnant, was used as the reference plasma. To optimize the plasma management, the two assays (TFPI resistance and TFPI activity) were simultaneously realized on the same aliquots after defrosting at 37°C for ten minutes.

### TFPI resistance

We used the assay described by tardy-Poncet *et al*. [11]TFPI resistance is defined as a poor anticoagulant response to TFPI. To evaluate the anticoagulant activity of exogenous TFPI, two modified diluted thromboplastin times (dTT) were determined, one with and one without exogenous TFPI. For this purpose, two reagents were prepared: R1 and R2. R1 comprised a Tris bovine serum antigen buffer (50 mmol L^-1^ Tris/HCl, 100 mmol L^-1^ NaCl, and 1 g L^-1^ of bovine serum albumin, pH 7.5) containing 50 mmol L^-1^ CaCl2 and tissue factor (Thromborel S, Behring, Paris, France), diluted to a final concentration of 1/150th. R1 is a referent dTT. R2 had the same composition as R1, except for the addition of purified human TFPI (American Diagnostica, Andresy, France), at a final concentration of 250 ng mL^-1^. R2 is a dTT with TFPI (final TFPI concentration in the reagent medium: 83 ng/mL). After a 15-s incubation of 50 mL of undiluted patient and control plasma samples at 37°C, clotting was initiated by adding 50 mL of the R1 solution, and the ‘dTT without TFPI’ was recorded. Identical plasma samples were then incubated with solution R2 to determine the ‘dTT with TFPI’. Clot formation was recorded for 240 s on a coagulometer (BCT Behring). All measurements were performed in duplicate and the final result was considered the mean of the two measurements. Detection of thrombus formation was made by optic detection at 405 nm.

The variation coefficient authorized for this measurement is 5%. TFPI sensitivity ratios were obtained by dividing the ‘dTT with TFPI’ by the ‘dTT without TFPI’. For each series, we also measured the TFPI sensitivity ratios of the reference plasma. The TFPI sensitivity ratio of the reference plasma was 2.01 (mean value of 30 measurements). The normalized ratio was then calculated by dividing the TFPI sensitivity ratio obtained for each of the patient and control plasma samples by the TFPI sensitivity ratio obtained for the reference plasma.

### TFPI activity assay

We used the assay described by Sandset *et al*. [18]: the TFPI activity assay is based on the ability of TFPI in the sample, with presence of FXa, to inhibit TF/FVIIa catalytic activity. The plasma is incubated in the presence of tissue factor, FXa, and FVIIa, which permit formation of TF/FVIIa/TFPI/FXa. The residual TF/FVIIa catalytic activity is measured by adding FX and specific Xa chromogenic substrate.

The recombinant Tissue Factor (Dade-Innovin) was purchased from Dade Behring Siemens (Saint Denis, France). The recombinant FVIIa (Novoseven 60KU) was provided by Novo Nordisk (Danemark). The bovine factor Xa (70 nkats), the bovine factor X (100μg), and the chromogenic substrate CS-11 (kit biophen heparin 6) were purchased from Hyphen Biomed (Neuville sur Oise, France). The polymerization fibrinogen inhibitor, Pefabloc FG 50 mg (Pentapharm) was distributed by Cryopep (Montpellier, France). The combined reagent is prepared in the ice, by dilution Tris bovine serum antigen buffer (50 mmol L^-1^ Tris/HCl, 100 mmol L^-1^ NaCl and 1 g L^-1^ of bovine serum albumin, pH 7.5), with TF (last dilution 1/100), CaCl2 10 mmol L^-1^, Pefabloc (100 μG/ml), FXa (1.3 nmol L^-1^), FVIIa (10 nmol L^-1^) (last concentrations in the measurement). The plasma samples were defrosted at 37°C for 10 minutes and had to be used within two hours. We took 25 μl from a 1/80 dilution in buffer TBS/BSA of each sample. A calibration range was made by six measurement points (0 to 150%) established by the reference plasma and the control plasma. After adding 100 μL from combined reagent, the incubation at 37°C for 30 minutes was realized, at which point 50 μl of a substrate reagent composed of bovine FX (0.2 U/mL) and chromogenic substrate (2.5 mg/mL) were added for a second incubation at 37°C for 30 minutes. The absorbance at 405 nm was measured by a spectrophotometer (Multiskan Ascent, Thermolab System), and a calibration curve was automatically drawn. TFPI activity was expressed as a percentage compared to the control group values.

### Statistical analysis

Statistical analysis was performed using SAS 9.4 and R statistical software, version 3.2.1. Qualitative data were described by absolute and relative frequencies (expressed in %) and were compared with Fisher exact test. Quantitative variables were described by mean and standard deviation and interquartile range and were compared by Student's t test. In the event of a non-normally distributed variable (assessed by Shapiro-Wilk test), a Wilcoxon Mann-Whitney test was performed. TFPI resistance and activity at each of the six visits (at 20, 24, 28, 32, and 36 weeks’ gestation, and post-partum) in patients with placenta-mediated adverse pregnancy outcomes and patients without were compared. TFPI plasma levels were summarised in boxplots for each of the five visits (20, 24, 28, 32 and 36 weeks) and were compared to postpartum levels using the Wilcoxon rank test. Tests were performed at the adjusted two-sided alpha level of 1%.

## Results

### Description of the study population

Between June 2008 and October 2010, two hundred pregnant women were included in the study, which enabled the analysis of 934 plasma samples. All samples were collected prior to the placenta-mediated adverse pregnancy outcome. TFPI assays were realized on 72 patients included in the study at Saint Etienne, at different stages of pregnancy, as well as in the postpartum period. Demographics and inclusion criteria are summarized in Table 1. During the study, 15 of the 72 patients had placenta-mediated adverse pregnancy outcomes with four PE, eight IUGR, and three associations between PE and IUGR. There was significantly more diabetes and history of placenta-mediated adverse pregnancy outcomes among patients with placenta-mediated adverse pregnancy outcomes compared to patients without. However, other demographic characteristics and inclusion criteria were not statistically different between patients with or without placenta-mediated adverse pregnancy outcomes.

**Table 1:** Patient characteristics at inclusion^a^.

	Total No = 72		Placenta-mediated adverse pregnancy outcomes No = 15		No placenta-mediated adverse pregnancy outcome No = 57		P value
Demographic data: mean ± standard deviation median (Q1;Q3)	mean ± SD	median (Q1-Q3)	mean ± SD	median (Q1-Q3)	mean ± SD	median (Q1-Q3)	
Age (years)	31.1 ±4.6	30.8(27.6−33.9)	32.5 ±4.2	32.5(28.2−34.5)	30.7 ±4.7	30.3(27.1−33.7)	0.19*
Gravidity	2.6 ± 1.9	2.0(1.0−4.0)	3.5 ± 2.4	3.0(1.0−5.0)	2.4 ± 1.7	2.0(1.0−3.0)	0.12**
Parity	1.1 ± 0.9	1.0(1.0−2.0)	1.1 ± 0.7	1.0(1.0−2.0)	1.1 ± 0.9	1.0(0.5−2.0)	0.92**
BMI (Kg/m^2^)	25.5 ±6.1	23.5(20.9−28.9)	25.2 ±6.2	24.2(20.4−27.7)	25.5 ±6.2	23.4(21.1−29.1)	0.85**
**Inclusion criteria**	**n**	**%**	**n**	**%**	**n**	**%**	
BMI>30 kg/m^2^	15	21.1	2	13.3	13	23.2	0.40***
Smoking	2	2.8	1	6.7	1	1.8	0.31***
Diabetes	5	7.0	3	20.0	2	3.6	0.03***
Kidney disease	0	0.0	0	0.0	0	0.0	0***
Hypertension	4	5.6	1	6.7	3	5.4	0.84***
Lupus	3	4.2	0	0.0	3	5.4	0.36***
Antiphospholipid Syndrome	3 ^b^	4.3	0	0.0	3	5.5	0.35***
Personal history of venous thromboembolism	15	21.1	1	6.7	14	25.0	0.12***
Personal history of PVP	47	66.2	15	100.0	32	57.1	0.002***
Familial history	18	25.4	3	20.0	15	26.8	0.59***

^a^data are available for 71 patients

^b^ data available for 70 patients

* Student’s t test

** Wilcoxon Mann-Whitney test

*** Fisher exact test

No.: number of; BMI: Body Mass Index; VTE: venous thromboembolism; SD: standard deviation; Q1: 1^st^ quartile; Q3: 3^rd^ quartile

### TFPI resistance

The threshold value of the normalized ratio (NR) TFPI has been defined by a reference pool of 34 controls, and has been determined at 0.70 (minimum value). The control group consisted of healthy volunteer’s women who were not using oral contraceptives and who were not pregnant. A value below 0.70 was associated with a TFPI resistance.

**Fig 1:**
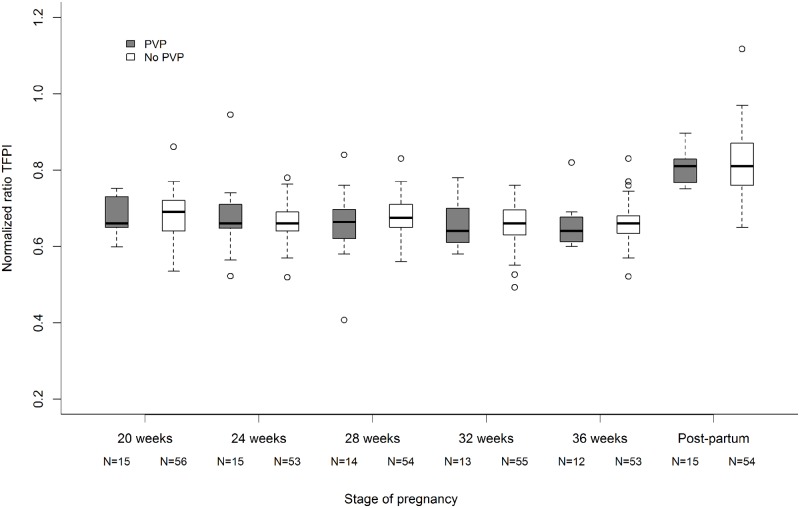
No difference between TFPI resistance (median normalized ratio) during pregnancy and postpartum for patients with placenta-mediated adverse pregnancy outcomes (PVP) and patients without (No PVP). The lower and upper limits of the box represent the first and third quartiles respectively and the heavy line inside the box the median. The horizontal line at the bottom and at the top of each plot represent the value of the lower and upper quartiles respectively. The circles represent the points that fall outside the nominal range of the data inferred from the upper and lower quartiles.

There was no difference regarding the normalized ratios TFPI between patients with and without placenta-mediated adverse pregnancy outcomes during pregnancy or during the postpartum period (Fig 1).

**Fig 2:**
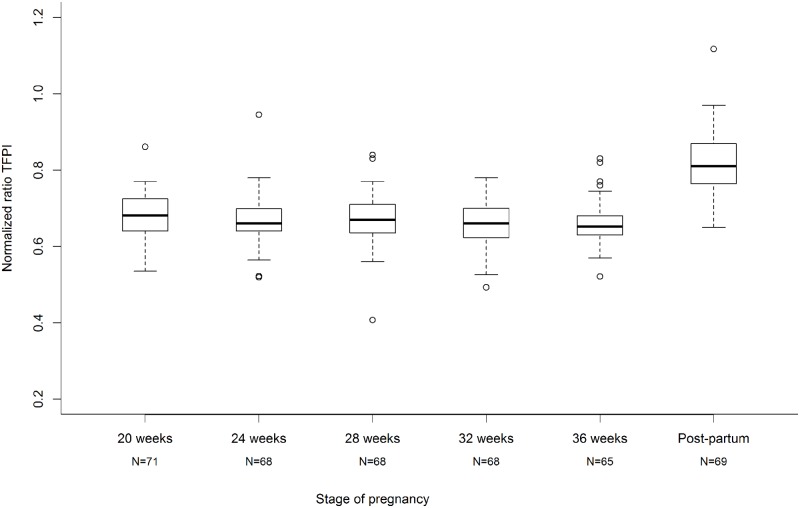
Median normalised ratio was below 0.70 during pregnancy and higher in postpartum. The lower and upper limits of the box represent the first and third quartiles respectively and the heavy line inside the box the median. The horizontal line at the bottom and at the top of each plot represent the value of the lower and upper quartiles respectively. The circles represent the points that fall outside the nominal range of the data inferred from the upper and lower quartiles.

For all patients, the normalised ratio of TFPI was constant and below 0.70 during pregnancy in every stage. It became normal in postpartum with a median normalized ratio of 0.81 globally (Fig 2). The differences between any stage of pregnancy and postpartum were constant regardless of pregnancy stage (Table 2).

**Table 2:** Evolution of TFPI resistance and activity during pregnancy and postpartum.

Gestational Age	Normalised ratio of TFPI		Comparison with postpartum period	TFPI activity %		Comparison with postpartum period
	mean ± SD	median (Q1−Q3)	*Absolute* difference 95% CI	mean ± SD	median (Q1−Q3)	*Absolute* difference 95% CI
**20 weeks**	0.68 ± 0.06	0.68 (0.64–0.73)	−0.14 (−0.16;−0.12)	78.0 ± 19.9	76.5 (66.2–91.1)	−8.2 (−15.2; −1.1)
**24 weeks**	0.67 ± 0.06	0.66 (0.64–0.70)	−0.15 (−0.18;−0.13)	79.9 ± 20.2	76.1 (68.5–90.3)	−6.2 (−13.5; 1.0)
**28 weeks**	0.67 ± 0.07	0.67 (0.64–0.71)	−0.15 (−0.17;−0.12)	83.2 ± 22.4	80.2 (69.7–94.1)	−2.9 (−10.5; 4.8)
**32 weeks**	0.66 ± 0.05	0.66 (0.62–0.70)	−0.16 (−0.18;−0.13)	85.9 ± 23.3	84.7 (70.6–100.9)	−0.4 (−8.2; 7.4)
**36 weeks**	0.66 ± 0.05	0.65 (0.63–0.68)	−0.16 (−0.19;−0.14)	88.8 ± 22.9	84.9 (77.8–100.6)	2.9 (−5.1; 10.9)
**Postpartum period**	0.82 ± 0.08	0.81 (0.76–0.87)	_	85.9 ± 22.3	82.9 (72.2–97.7)	_

### TFPI activity

**Fig 3:**
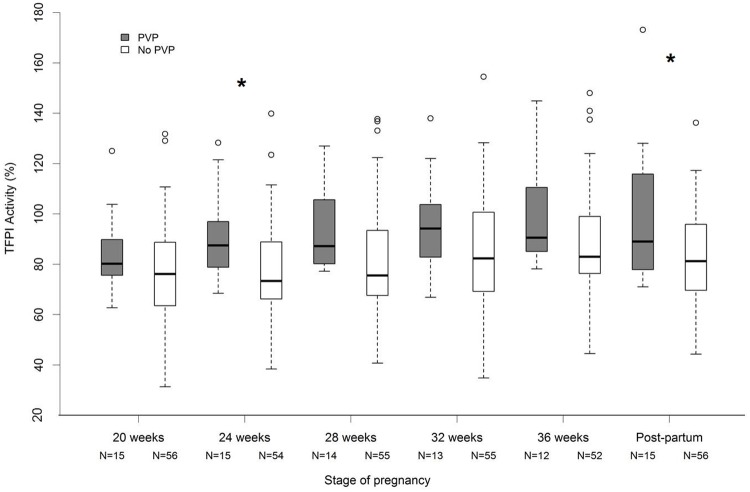
Patients with placenta-mediated adverse pregnancy outcomes (PVP) showed higher median TFPI activity values than patients without (No PVP) both during pregnancy and in the postpartum period. The lower and upper limits of the box represent the first and third quartiles respectively and the heavy line inside the box the median. The horizontal line at the bottom and at the top of each plot represent the value of the lower and upper quartiles respectively. The circles represent the points that fall outside the nominal range of the data inferred from the upper and lower quartiles. * p<0.05, Wilcoxon Mann-Whitney test. PVP for Placental Vascular Pathology.

TFPI activity values were higher in patients with placenta-mediated adverse pregnancy outcomes than in patients without during pregnancy. This difference was statistically significant at 24 weeks and close to statistical significance at 28, 32, and 36 weeks. During the postpartum period, patients with placenta-mediated adverse pregnancy outcomes showed higher TFPI activity values than patients without and this difference was statistically significant (*p* = 0.04). (Table 3, Fig 3).

**Table 3:** Comparison of TFPI activity during pregnancy in patients with placenta-mediated adverse pregnancy outcomes and without.

Gestational Age	Placenta-mediated adverse pregnancy outcomes		No placenta-mediated adverse pregnancy outcome		*Absolute difference* *95% CI*	*P value*
	mean ± SD	median (Q1−Q3)	mean ± SD	median (Q1−Q3)		
**20 weeks**	84.6 ± 15.5	80.2 (74.9−91.7)	76.2 ± 20.6	76.2 (63.6−88.7)	8.3 (-1.2; 17.9)	0.15*
**24 weeks**	90.2 ± 17.0	87.5 (78.6−98.3)	77.0 ± 20.3	73.4 (66.2−88.9)	13.2 (3.0; 23.3)	0.02*
**28 weeks**	93.1 ± 16.7	87.2 (80.2−105.6)	80.6 ± 23.1	75.5 (66.8−94.1)	12.5 (1.8; 23.1)	0.06*
**32 weeks**	96.6 ± 19.1	94.2 (82.8−103.7)	83.4 ± 23.6	82.3 (68.2−100.8)	13.2 (1.1; 25.3)	0.06*
**36 weeks**	98.6 ± 20.3	90.6 (85.1−110.5)	86.6 ± 23.1	83.0 (76.4−99.0)	12.0 (-1.1; 25.1)	0.10*
**Postpartum period**	98.6 ± 27.8	89 (75.8−116.4)	82.5 ± 19.6	81.2 (69.7−95.9)	16.1(1.1; 31.1)	0.04*

* Wilcoxon Mann-Whitney test

No.: number of; SD: standard deviation; Q1: 1^st^ quartile; Q3: 3^rd^ quartile

For high-risk population studied, TFPI activity was significantly decreased at 20 and 24 weeks of pregnancy compared with that in the postpartum period (*p* < 0.0001 and *p* = 0.0005 at 20 and 24 weeks, respectively). Significant difference was not found in the rate of TFPI activity at 28 weeks compared with that in the postpartum period (Table 2).

## Discussion

Our results showed that pregnancy induces TFPI exogenous resistance without any difference between patients with and without placenta-mediated adverse pregnancy outcomes. Furthermore, there was an increase in TFPI activity during pregnancy at 24, 28 and 32 weeks, as well as in the postpartum period in patients who experienced placenta-mediated adverse pregnancy outcomes compared to patients who did not, and this difference was present prior to the occurrence of placenta-mediated adverse pregnancy outcomes.

### Revealing TFPI resistance

TFPI exogenous resistance is involved in physiological hypercoagulability, inherent in gravid state, caused by acquired protein S deficiency and resistance to activated protein C (not associated with a factor V Leyden mutation) [19]. Some studies suggest a concomitant action of TFPI and protein S. Hackeng *et al*. [10] showed inhibition of thrombin generation by the TFPI / protein S system. Castoldi *et al*. [9] noted that protein S deficiency was more frequently associated with a reduced rate of TFPI. Tardy-Poncet *et al*. reports TFPI NR (and PS activity) measured in plasma of 7 women during pregnancy and 3 months after delivery. Median TFPI NR values were statistically significantly lower during pregnancy than 3 months after delivery [20]. Our study also highlights the possibility of increased resistance to exogenous TFPI during pregnancy on a larger population.

### TFPI activity assay

Our results demonstrated that the rate of TFPI activity was significantly higher overall in patients with placenta-mediated adverse pregnancy outcomes. We believe that these higher TFPI values match an overexpression of tissue factor, which is known to increase during pregnancy, and may correlate in particular to placenta-mediated adverse pregnancy outcomes.

Several studies support this hypothesis. Godoi *et al*. [21] showed significantly higher TFPI levels in pre-eclamptic women compared to those in normotensive pregnant patients and non-pregnant patients, for which the results were significant before 34 weeks. No significant differences in tissue factor rate were found between the three groups, and the tendency for TFPI to increase in late pregnancy was demonstrated. Erez *et al*. [22] reported the same results, distinguishing between placenta-mediated adverse pregnancy outcomes that results in foetal growth restriction and in pre-eclampsia. TF concentrations were increased in both populations significantly, compared to those in pregnant women without placenta-mediated adverse pregnancy outcomes, though TFPI concentrations were increased in pre-eclamptic patients only, with no significant difference in the foetal growth restriction group compared to the healthy pregnant women group. Thus, TFPI / TF ratio was significantly decreased in the PE group and increased in the foetal growth restriction group. The authors hypothesized placenta-mediated adverse pregnancy outcomes (maternal or foetal) were phenotypically expressed. Abdel Gader *et al*. [23] compared total and free TFPI levels and found that pre-eclamptic patients show increased free TFPI levels compared to normal pregnancy patients and those with gestational hypertension during pregnancy and postpartum (24 hours after birth). The differences were significant. Ittel *et al*. [15] found higher concentrations of free TFPI in non-pregnant patients compared to pregnant patients (with a normal pregnancy) in the 1^st^, 2^nd^, and 3^rd^ trimester of pregnancy. Moreover, during the period of at least 12 weeks postpartum, they found that concentrations of TFPI were significantly lower among patients who experienced a gestational vascular complication compared to non-pregnant patients, which could correspond to a potential indicator of placenta-mediated adverse pregnancy outcomes risk.

Other studies found decreased levels of TFPI in patients with placenta-mediated adverse pregnancy outcomes: Aharon *et al*. [24] showed significantly decreased TFPI levels in patients with placenta-mediated adverse pregnancy outcomes during pregnancy. Furthermore, the TF / TFPI ratio was increased in this population, emphasizing the hypercoagulable mechanism in the genesis of PE. They added that the decrease could be palliated by a LMWH treatment, thereby increasing the rate of TFPI. Teng *et al*. [7] demonstrated similar results.

Normalized ratio of TFPI is hypersensitive to analytical test preconditions, with the risk that traces of TF are present in the sample and can alone be responsible for a decrease in the ratio.

## Conclusion

Our study shows that the normalized TFPI ratio is not a discriminating factor between patients with placenta-mediated adverse pregnancy outcomes and without, although there were significantly higher rates of TFPI activity in patients with placenta-mediated adverse pregnancy outcomes than in patients without. Similar results are also described in the literature. Thus, the increase in TFPI levels in PVP patients could match the overexpression of tissue factor (TF). A study combining clinical, sonographic, and dosage of angiogenic factors data for the prediction of placenta-mediated adverse pregnancy outcomes in high-risk patients seems necessary to establish an effective screening strategy.

## Supporting information

S1 Table All available data. (PDF)
