# Correction: Tissue factor pathway inhibitor for prediction of placenta-mediated adverse pregnancy outcomes in high-risk women: AngioPred study

**DOI:** 10.1371/journal.pone.0181474

**Published:** 2017-07-12

**Authors:** Aurélie Di Bartolomeo, Céline Chauleur, Brigitte Tardy, Michèle Piot, Jean-Christophe Gris, Céline Chapelle, Edouard Noblot, Silvy Laporte, Tiphaine Raia-Barjat

Drs. Brigitte Tardy and Michèle Piot are not included in the author byline. Brigitte Tardy should be listed as the third author. Michèle Piot should be listed as the fourth author. Brigitte Tardy’s contributions to the manuscript are as follows: Conceived and designed the experiments, conducted formal analysis. Michèle Piot’s contributions to the manuscript are as follows: Conceived and designed the experiments, contributed to the investigation of the manuscript. The correct author byline and affiliations are as follows:

Aurélie Di Bartolomeo^1^, Céline Chauleur^1,2^, Brigitte Tardy^2,3^, Michèle Piot^2,3^, Jean-Christophe Gris^4,5^, Céline Chapelle^2,6^, Edouard Noblot^1^, Silvy Laporte^2,6^, Tiphaine Raia-Barjat^1,2^.

**1** Department of Gynaecology and Obstetrics, University Hospital, Saint Etienne, France. **2** INSERM UMR 1059, Saint Etienne University Jean Monnet Saint Etienne France. **3** Laboratory of Haematology, University Hospital, Saint Etienne, France. **4** Laboratory of Haematology, University Hospital, Nîmes, France. **5** Research Unit EA2992, Montpellier University, Montpellier, France. **6** Clinical Pharmacology Unit, University Hospital, Saint Etienne, France.

The correct citation is: Di Bartolomeo A, Chauleur C, Tardy B, Piot M, Gris J-C, Chapelle C, et al. (2017) Tissue factor pathway inhibitor for prediction of placenta-mediated adverse pregnancy outcomes in high-risk women: AngioPred study. PLoS ONE 12(3): e0173596. https://doi.org/10.1371/journal.pone.0173596
