# Supplementary material for: Tissue factor pathway inhibitor for prediction of placenta-mediated adverse pregnancy outcomes in high-risk women: AngioPred study
Source: PLoS One. 2017 Mar 22;12(3):e0173596. doi: 10.1371/journal.pone.0173596 (PMC5362074; doi:10.1371/journal.pone.0173596)
Supplement: S1 Table — (PDF) [file pone.0173596.s001.pdf]

ID	IDP_centre	Age	VIS_taille	VIS_poids	BMI	VIS_gestit_
51	1	39	155	90	37,46	3
74	1	32	168	49	17,36	1
101	1	32	161	53	20,45	5
116	1	31	158	52	20,83	3
120	1	32	158	50	20,03	1
121	1	28	160	70	27,34	1
144	1	28	160	71	27,73	4
149	1	36	165	66	24,24	6
174	1	27	165	55	20,20	4
56	1	26	159	73	28,88	2
93	1	34	160	59	23,05	8
97	1	33	170	71	24,57	2
13	1	40	163	103	38,77	8
106	1	29	163	58	21,83	1
141	1	33	155	61	25,39	3
45	1	29	168	63	22,32	1
46	1	33	166	100	36,29	3
49	1	42	168	60	21,26	2
50	1	32	168	61	21,61	5
53	1	30	166	62	22,50	3
58	1	34	163	73	27,48	2
59	1	27	157	60	24,34	1
73	1	26	162	85	32,39	0
84	1	38	178	72	22,72	8
88	1	30	163	55	20,70	1
91	1	28	164	74	27,51	3
99	1	33	177	103	32,88	2
103	1	26	162	73	27,82	2
109	1	40	169	49	17,16	1
110	1	27	167	65	23,31	1
115	1	34	163	121	45,54	4
118	1	24	170	92	31,83	3
122	1	27	158	46	18,43	1
129	1	38	170	58	20,07	1
130	1	33	166	50	18,14	2
131	1	27	177	71	22,66	1
132	1	30	168	64	22,68	2
135	1	34	160	80	31,25	6
136	1	29	159	51	20,17	3
139	1	30	160	79	30,86	2
142	1	35	171	61	20,86	5
143	1	24	167	75	26,89	6
155	1	25	160	51	19,92	2
160	1	27	165	95	34,89	2
161	1	36	160	65	25,39	5
166	1	35	164	59	21,94	5
175	1	32	163	65	24,46	2
189	1	31	155	55	22,89	3

190	1	32	179	60	18,73	1
192	1	23	160	56	21,88	5
194	1	29	157	57	23,12	1
182	1	31	160	100	39,06	2
197	1					
180	1	27	161	64	24,69	2
179	1	26	166	74	26,85	1
37	1	31	166	66	23,95	2
41	1	28	166	85	30,85	2
44	1	26	156	47	19,31	2
48	1	33	167	58	20,80	4
57	1	39	157	93	37,73	1
60	1	32	164	62	23,05	6
72	1	25	175	64	20,90	2
75	1	38	169	48	16,81	4
77	1	24	162	100	38,10	1
81	1	28	163	51	19,20	0
85	1	34	170	85	29,41	3
90	1	30	166	70	25,40	1
78	1	29	170	68	23,53	2
89	1	25	158	80	32,05	1
38	1	32	164	64	23,80	1
94	1	21	173	67	22,39	1
32	1	26	159	73	28,88	1

VIS_parit_	VIS_tabac_cig_j	PVP	V20_fact_hemo_DPS_TTD_Tp	V20_fact_hemo_DPS_TTD_TFPi	V20_fact_hemo_DPS__ratio	V20_fact_hemo_DPS_ratio_norm
1	0	0	45,9	95,5	2,080610022	0,74
1	0	0	54,6	101,8	1,864468864	0,66
1	0	0	61,8	129,5	2,095469256	0,77
0	0	0	55,8	104,8	1,878136201	0,69
1	0	1	58,5	112,2	1,917948718	0,66
1	0	0	56,1	107,6	1,918003565	0,71
2	3	0	59,1	100,9	1,707275804	0,63
2	0	0	78,5	139,4	1,775796178	0,66
0	0	0	59,9	115,4	1,92654424	0,71
0	0	1	55,2	107,4	1,945652174	0,73
2	0	0	64,2	125	1,947040498	0,73
1	0	0	57,3	108,3	1,890052356	0,7
1	0	0	60,1	112,3	1,868552413	0,7
1	0	0				
2	0	1	55,9	106,6	1,906976744	0,65
1	0	0	48,7	98	2,012320329	0,71
2	0	0	62	110,3	1,779032258	0,64
0	0	0	32,4	64,7	1,99691358	0,7
3	0	0	56,6	110,3	1,948763251	0,68
3	0	1	56,9	101,3	1,780316344	0,64
2	0	0	91	196,2	2,156043956	0,77
1	0	1	56,2	102,2	1,818505338	0,66
0	0	1	41,1	87,2	2,121654501	0,73
2	0	0	59,6	131,4	2,204697987	0,74
0	0	0	56,6	103,2	1,823321555	0,61
3	0	0	60,5	107	1,768595041	0,66
1	0	0	53,9	110	2,040816327	0,7
2	0	0	52,1	99,4	1,907869482	0,71
0	0	0	59,1	113,5	1,920473773	0,68
1	0	0	55,8	95,3	1,707885305	0,6
3	0	0	66,3	116,2	1,752639517	0,65
2	0	0	61	97,2	1,593442623	0,59
0	0	0	66,3	120,5	1,817496229	0,66
0	0	1	54,9	98,1	1,786885246	0,65
1	0	1	49,7	96,5	1,941649899	0,66
0	0	0	53,3	113,8	2,135084428	0,72
1	0	0	90,9	152,2	1,674367437	0,58
3	0	0	57,1	97,4	1,705779335	0,6
0	0	0	53,9	109,2	2,025974026	0,71
1	0	1	57,9	117	2,020725389	0,73
2	0	0	53,8	113	2,100371747	0,7
1	0	0	67,1	120,2	1,791356185	0,6
0	0	0	57,3	107,4	1,87434555	0,64
1	0	0	59,2	118	1,993243243	0,72
2	0	0	62,3	108,1	1,735152488	0,63
1	0	0	65,3	117,9	1,805513017	0,65
1	0	0	67,1	116,6	1,737704918	0,61
0	0	0	56,8	117,7	2,072183099	0,73

1	3	0	62,7	133,1	2,122807018	0,73
1	0	0	57,8	118,4	2,048442907	0,73850878
1	0	0	54,4	118,7	2,181985294	0,736809435
1	0	0	57,5	112,9	1,963478261	0,640873701
		0	52,8	96,2	1,821969697	0,63
1	0	1	55	106,7	1,94	0,651764706
0	0	0	51,7	92,6	1,791102515	0,611902839
1	0	0	60	138,6	2,31	0,7104
1	0	0	65,3	175,4	2,686064319	0,861038039
2	0	0	64,1	134,9	2,104524181	0,728588335
2	0	0	70,7	108,9	1,540311174	0,535427844
1	0	0	56,2	123,5	2,197508897	0,758614704
2	0	0	53,2	102,4	1,92481203	0,652437849
0	0	0	102,5	217,8	2,124878049	0,739830762
0	0	0	52,2	102,8	1,969348659	0,691301653
1	0	0	56,8	102,9	1,811619718	0,597120095
0	0	0	56,7	117,1	2,065255732	0,680719959
2	0	1	52,6	114,1	2,169201521	0,73738991
1	0	0	52,4	108,9	2,078244275	0,706470258
2	0	1	57,5	103	1,791304348	0,601058146
1	0	1	58,6	112,3	1,916382253	0,598801284
1	0	0	74,6	153	2,050938338	0,640845275
1	0	1	48,7	97,8	2,008213552	0,6933413
1	0	1	56,3	122,6	2,177619893	0,75182931

V20_fact_hemo_DPS_TTD_TFPi_act	V24_fact_hemo_DPS_TTD_Tp	V24_fact_hemo_DPS_TTD_TFPi	V24_fact_hemo_DPS__ratio	V24_fact_hemo_DPS_ratio_norm	V24_fact_hemo_DPS_TTD_TFPi_act	V28_fact_hemo_DPS_TTD_Tp
	47,5	100,9	2,124210526	0,75	63,43	53
49,96	54	99,2	1,837037037	0,65	38,42	56,3
85,6	60,3	96,6	1,60199005	0,59	69,37	69,9
78,87	57,2	109,7	1,917832168	0,71		60,9
74,9	61,6	119,3	1,936688312	0,67	79	62,6
76,2	53,7	95,2	1,772811918	0,65	69,1	61,3
61,9	59,2	106,5	1,798986486	0,66	68,1	53,5
73,6	76,4	136,2	1,782722513	0,66	78,5	66,5
31,3	57,1	103,5	1,812609457	0,67	49,1	57
91,7	52,2	91,7	1,756704981	0,66	80,1	52,6
92,2	64	113,7	1,7765625	0,67	111,4	60,7
78,4	59,3	124,1	2,092748735	0,78	66,8	56
70,5	60,8	113,2	1,861842105	0,69	70,8	58,4
76,5					73	
69,7	57	106,1	1,861403509	0,64	75,2	
68,8	51,8	95,4	1,841698842	0,65	71,6	46,4
70,4	56,7	97,9	1,726631393	0,62	72,6	54,8
59	33,2	61,3	1,846385542	0,64	66,2	35,6
62,2	56,8	93	1,637323944	0,57	63,6	59,5
98,5	55,6	99,5	1,789568345	0,65	95,6	55,6
35,4	81,2	134	1,650246305	0,59	54,1	89,3
62,7	61,7	112,3	1,820097245	0,66	68,5	62,1
80,2	56,4	117,6	2,085106383	0,71	87,5	53,5
60,3	57,6	112,2	1,947916667	0,65	66,2	59,8
74	53,9	98,2	1,821892393	0,61	69,9	56,5
95,7	60,3	107,1	1,776119403	0,67	101,9	55
80						55,9
94,1	48,5	85,3	1,758762887	0,66	81,7	50,4
76,2	60	111	1,85	0,65	73,9	57,9
102,9	56,6	96,1	1,697879859	0,6	110,3	62,8
76,7	67,6	116,3	1,720414201	0,64	69,7	72,7
100,8	63,8	110,3	1,728840125	0,64	98,9	67,1
131,8	67,1	119,3	1,777943368	0,65	139,9	64,9
81,8	59,3	121	2,040472175	0,74	93,8	59,6
71,9	51,9	109,3	2,105973025	0,71	78,6	56,2
84,4	54,4	114,5	2,104779412	0,71	92,2	55,6
129,1	88,1	157,3	1,785471056	0,62	123,5	95,9
96,2	57	111,7	1,959649123	0,69	73,7	54,1
108,3	54	102,1	1,890740741	0,66	111,5	53,7
86,4	59,2	119,9	2,025337838	0,74	100,7	57,5
93,8	56	120	2,142857143	0,71	107	57
100,3	65,8	119,5	1,816109422	0,61	94,2	66,3
61,2	55,1	100,7	1,827586207	0,62	56,5	56,5
57,5						59,7
42,6	63	111	1,761904762	0,64	63,9	67,8
85,3	59,5	113,4	1,905882353	0,69	83,3	60,1
66,7	61,8	110,6	1,789644013	0,63	70,8	55,7
66,2	59,3	121,8	2,053962901	0,73	61,4	50

61,9	66,1	128,8	1,948562784	0,67	48,7	70,5
91,1	60,7	123,9	2,041186161	0,735892564	88,9	52,5
46,6	56,5	127,7	2,260176991	0,763213087	45,4	59,8
40,5	59,1	120,7	2,042301184	0,666601278	48,4	60,9
69,1	53,1	90,6	1,706214689	0,59	77,5	53,9
80,2	57,9	119,3	2,06044905	0,692230912	80	60,8
64,9	52,3	102,3	1,956022945	0,668245387	81,5	
96,8	61,3	148,6	2,424143556	0,74550285	90	53,4
55,2	61,3	135,8	2,215334421	0,710142044	52,3	60,4
86,3	64,6	127,5	1,973684211	0,683291409	89,6	64,7
66,3	63,8	95,2	1,492163009	0,518691052	74,9	
76,1	53,3	116,5	2,185741088	0,754552271	70,2	54,5
66,2	53,1	103,5	1,949152542	0,660688354	60,7	54,8
65,3	>120	>250	.	.	76,7	116,2
80,4	57	109,6	1,922807018	0,674964112	76,1	57,1
75,1	59,3	116,1	1,957841484	0,645315616	74,3	55,2
83	57,9	118,4	2,044905009	0,674012245	83,1	53,6
125	51,9	100,2	1,930635838	0,656292822	128,3	52,1
110,7	53,8	99,6	1,851301115	0,629324085	103,5	55
103,8	57,1	109,7	1,921190893	0,644640559	121,5	50,8
77,25	62,4	112,6	1,804487179	0,563838054	75,7	48,7
78,35	69,6	157	2,255747126	0,704840736	80,9	63,1
88	64,8	98	1,512345679	0,522141541	90,3	64,1
76,4	47,5	130	2,736842105	0,94490233	98,3	58,8

V28_fact_hemo_DPS_TTD_TFPi	V28_fact_hemo_DPS__ratio	V28_fact_hemo_DPS_ratio_norm	V28_fact_hemo_DPS_TTD_TFPi_act	V32_fact_hemo_DPS_TTD_Tp	V32_fact_hemo_DPS_TTD_TFPi	V32_fact_hemo_DPS__ratio
84,5	1,594339623	0,57	68,4	59	99,3	1,683050847
120,4	2,138543517	0,76	46,06	57,2	119,1	2,082167832
125,7	1,798283262	0,66	75,51	56	105,5	1,883928571
119,9	1,968801314	0,73	68,64	57,8	103,5	1,790657439
138,4	2,21086262	0,76	80,2	66,4	123,1	1,853915663
117,9	1,923327896	0,71	51,6	58,6	108,3	1,848122867
92	1,719626168	0,63	75,7	54,2	91,8	1,693726937
118,2	1,777443609	0,66	75	70,4	121,4	1,724431818
102,5	1,798245614	0,66	53,9	59,4	106,7	1,796296296
92	1,74904943	0,66	85,8	54,3	113,3	2,086556169
110,6	1,822075783	0,68	89,8	62,7	117,7	1,877192982
107,3	1,916071429	0,71	78,1	60,2	115,1	1,911960133
106,8	1,828767123	0,68	73,7	57,8	97,7	1,690311419
			69,7			
84,3	1,816810345	0,64	66,3	37,5	67,3	1,794666667
89,4	1,631386861	0,58	80,9	86,2	160,4	1,860788863
65,8	1,848314607	0,65	59,6	57,8	116,1	2,008650519
121,2	2,03697479	0,71	75,8	56,7	103,1	1,818342152
96,6	1,737410072	0,63	110,9	56,8	95,9	1,688380282
175,1	1,960806271	0,7	48,3	75,7	136,8	1,807133421
100,1	1,611916264	0,58	77,2	68,5	122,6	1,789781022
130,8	2,444859813	0,84	78,5			
119	1,989966555	0,67	69,9	59,7	127	2,127303183
114,9	2,033628319	0,68	80,5	57,6	113,8	1,975694444
94,9	1,725454545	0,65	102,1	62,3	118,7	1,90529695
122	2,182468694	0,75	92,8	57,2	104,5	1,826923077
99,5	1,974206349	0,74	70,8	51,8	95,4	1,841698842
111,7	1,929188256	0,68	74,2	57,3	111,5	1,945898778
120,2	1,914012739	0,67	133,1	55,8	102,3	1,833333333
122,5	1,685006878	0,63	66,8	68,2	112,1	1,643695015
117,3	1,748137109	0,65	122,4	61,7	109,8	1,779578606
110,5	1,702619414	0,62	136,8	64,4	118,5	1,840062112
104,3	1,75	0,64	89	58,9	113,6	1,928692699
103,8	1,846975089	0,62	88,6	53,8	97,4	1,810408922
111	1,996402878	0,67	85,6	52	101,2	1,946153846
171,6	1,789363921	0,62	137,7	92,3	163,2	1,768147346
119,3	2,205175601	0,77	107,8	58,4	108	1,849315068
110,1	2,05027933	0,72	111,8	56,1	114,9	2,048128342
120	2,086956522	0,76	105,6	58,5	119,7	2,046153846
126,3	2,215789474	0,74	114	57,9	115,9	2,001727116
119,8	1,80693816	0,6	109,6	65,2	115,1	1,765337423
113,8	2,014159292	0,69	69,1	54,2	110	2,029520295
117,2	1,963149079	0,71	60	60,4	125,8	2,082781457
105,3	1,553097345	0,56	89,4	64,5	108,4	1,680620155
113,8	1,893510815	0,68	85	61,3	119,8	1,954323002
111,6	2,003590664	0,71	94,7	62	111,7	1,801612903
117,4	2,348	0,83	65,7	58,1	118	2,030981067

134,6	1,909219858	0,66	55,6	67,4	130,5	1,93620178
88,5	1,685714286	0,607737124	101,9	51,7	107,7	2,083172147
126,9	2,122073579	0,716578539	41,2	56,9	111,1	1,95254833
124,3	2,041050903	0,66619319	40,7	56,1	103,2	1,839572193
117,2	2,174397032	0,75	72,1	50,2	96,5	1,922310757
122,6	2,016447368	0,677448055	79,9	58,4	100,8	1,726027397
				53,3	93,5	1,754221388
107,1	2,005617978	0,616792646	90,5	56,9	120,4	2,11599297
122,9	2,034768212	0,652260193	52,7	57,7	123,3	2,136915078
129,5	2,001545595	0,692937047	89,6	62,8	131,8	2,098726115
				71,3	107,8	1,511921459
105,1	1,928440367	0,665728007	69,9	58,6	123,8	2,112627986
105,2	1,919708029	0,650707787	61,2	57,6	110,7	1,921875
230,9	1,987091222	0,691856746	72,7	118,3	227,4	1,922231615
117,7	2,061295972	0,723577974	98	59,2	119,6	2,02027027
100,8	1,826086957	0,601888579	71,9			
94,8	1,768656716	0,582959247	94,1	51,7	77,4	1,497098646
104,5	2,005758157	0,68182961	127	55,2	111,6	2,02173913
112,8	2,050909091	0,697178042	103,5	55,4	96,4	1,740072202
101	1,988188976	0,667121241	122,85	54,6	101,6	1,860805861
63,5	1,303901437	0,407422817	92,9	61,8	116,3	1,881877023
144,4	2,288431062	0,715053303	83,1	55,1	97,2	1,764065336
110,4	1,722308892	0,594631923	82,8	62	110,7	1,785483871
118,5	2,015306122	0,69579003	82,7	61,2	129,7	2,119281046

V32_fact_hemo_DPS_ratio_norm	V32_fact_hemo_DPS_TTD_TFPi_act	V36_fact_hemo_DPS_TTD_Tp	V36_fact_hemo_DPS_TTD_TFPi	V36_fact_hemo_DPS__ratio	V36_fact_hemo_DPS_ratio_norm	V36_fact_hemo_DPS_TTD_TFPi_act
0,6		52,1	91	1,746641075	0,62	
0,74	36,91	52	96	1,846153846	0,66	45,38
0,69	116,11	57,9	91	1,571675302	0,58	80,97
0,66	107,54	52	91,4	1,757692308	0,65	
0,64	87,9					
0,68	63,7	59,8	108,5	1,814381271	0,67	76,1
0,62	78,9	51,9	79,7	1,535645472	0,57	82,2
0,64	70,2	66,1	115	1,7397882	0,64	79,1
0,66	34,8	54,2	87,7	1,618081181	0,6	44,5
0,78	82,8	55,1	102,1	1,852994555	0,69	78,2
0,7	101,3	56,5	99,1	1,753982301	0,66	104,5
0,71	74,8	59,9	108,3	1,808013356	0,67	78,1
0,63	71,1	54,4	103,5	1,902573529	0,71	70,4
	73,4					83,7
0,64	65,3	49,2	86,8	1,764227642	0,63	82,2
0,67	68,2	59,1	109,2	1,847715736	0,66	84,8
0,7	83,9	53,7	87,8	1,635009311	0,57	83,1
0,63	70,2	55,8	102,8	1,842293907	0,64	82,9
0,61	103,7	54,1	96,4	1,781885397	0,64	116,1
0,65	48,4	87,5	164,7	1,882285714	0,67	76,6
0,65	66,9	58,1	110,8	1,907056799	0,69	81,1
0,71	72,3	62,1	121,8	1,961352657	0,66	80,4
0,66	87,1	56,9	119,9	2,107205624	0,71	71,6
0,71	100,9	66,7	119,5	1,791604198	0,67	101,7
0,63	100,8	61	115,9	1,9	0,65	98,5
0,69	67,2	54,1	96,6	1,785582255	0,67	81,5
0,68	75,6	56,7	112	1,975308642	0,69	77,5
0,64	122,5	53,4	95,7	1,792134831	0,63	118,2
0,61	76,8	67,2	112,4	1,672619048	0,62	88,8
0,66	100,8	60,6	107	1,765676568	0,66	137,5
0,67	154,5	64,6	114	1,764705882	0,64	148
0,7	81,5	51,8	91,8	1,772200772	0,64	96,3
0,61	88,6	53,9	98,9	1,834879406	0,62	84,2
0,66	82,4	53,8	111,5	2,072490706	0,7	90,5
0,61	128,3	85,4	158	1,850117096	0,64	141
0,65	111,8	59,9	113,1	1,888146912	0,66	99,5
0,72	104,6	54,4	119,8	2,202205882	0,77	116,9
0,74	122	56,3	126,8	2,252220249	0,82	104,9
0,67	110	57,7	117,4	2,034662045	0,68	104
0,59	113	67,5	129,5	1,918518519	0,64	117
0,69	70,9	56,2	116,3	2,069395018	0,71	78,3
0,76	66,4	59,4	136,4	2,296296296	0,83	69,5
0,61	91,8	61,3	108	1,76182708	0,64	88,8
0,7	94	59,8	112	1,872909699	0,68	84,9
0,64	87,1	60	108,1	1,801666667	0,64	87,2
0,72	63,1	60,1	130	2,163061564	0,76	66,8

0,67	56,7	59,1	118,8	2,010152284	0,69	47,2
0,751029436	98,7	48,9	101,1	2,067484663	0,745373753	124
0,659333514	50,8	56,1	117,7	2,098039216	0,708462653	52,7
0,600431114	38,6	56,7	111,8	1,971781305	0,643583791	47
0,66	91,6	51,2	98,8	1,9296875	0,66	91,3
0,579878216	94,2	57,5	103,5	1,8	0,604730139	88,2
0,599302965	71	54	94,6	1,751851852	0,598493449	77,4
0,650736539	82,3	57,1	128	2,241681261	0,68938977	87,1
0,685004136	61,7	58,4	105,8	1,811643836	0,580736003	63,4
0,726581038	99,9	66,4	123,1	1,853915663	0,64182742	106,9
0,52555929	91,8	80,2	120,3	1,5	0,52141527	86,4
0,729312476	76	56,8	120,2	2,116197183	0,730544619	78,5
0,651442308	61,1	54	103,8	1,922222222	0,651560003	57,3
0,669274211	75,5	105	191,2	1,820952381	0,63401125	91,3
0,709176697	87					
0,493452173	100,5					
0,68726212	138	55,5	108,2	1,94954955	0,662722275	144,9
0,591513362	112	58,6	107,7	1,837883959	0,624763109	113,6
0,624378834	108,85	53,5	95,6	1,786915888	0,599585632	124,2333333
0,588019627	102,45	58,5	112,8	1,928205128	0,602495512	85,95
0,551207666	85,55	61,3	114	1,859706362	0,581092085	74,4
0,61644326	100,9	63,2	113,4	1,794303797	0,619488364	85,9
0,731687661	78,3	54,1	103,1	1,905730129	0,657958614	92,9

VPP_fact_hemo_DPS_TTD_Tp	VPP_fact_hemo_DPS_TTD_TFPi	VPP_fact_hemo_DPS__ratio	VPP_fact_hemo_DPS_ratio_norm	VPP_fact_hemo_DPS_TTD_TFPi_act
52,6	138,5	2,633079848	0,94	70,1
57,2	137,1	2,396853147	0,85	60,03
61,7	147,2	2,385737439	0,88	98,56
58,6	150,9	2,575085324	0,95	74,96
63,5	145,2	2,286614173	0,79	97
62,8	125,2	1,993630573	0,73	73,9
50,4	114,1	2,263888889	0,83	86,9
67,3	118,1	1,754829123	0,65	103,9
58,9	148,3	2,517826825	0,93	45,15
53	107,2	2,022641509	0,76	75,1
59,3	133,5	2,251264755	0,84	92,6
57,1	134,3	2,352014011	0,88	72,2
52,2	104,3	1,998084291	0,74	72,7
				64,7
58,2	130,8	2,24742268	0,763980106	89
52,1	119,3	2,289827255	0,81	81,5
61,9	124,1	2,004846527	0,72	66,3
58,9	156,5	2,65704584	0,93	58,7
56,2	134,9	2,400355872	0,84	77,4
57,1	121,7	2,131348511	0,77	117,8
				44,3
67,5	143,3	2,122962963	0,77	85,9
56,9	134,5	2,363796134	0,81	100
60,4	174,8	2,894039735	0,97	86,2
59,9	128,7	2,148580968	0,72	85,4
61	131,4	2,154098361	0,81	102,3
66,2	152	2,296072508	0,79	83,7
52	106,9	2,055769231	0,77	88,2
53,5	122	2,280373832	0,8	71,3
59,2	135,7	2,29222973	0,81	115,4
69,3	138,1	1,992784993	0,74	80,6
62,4	126,3	2,024038462	0,75	107,8
62,8	138,8	2,210191083	0,8	117,3
54,8	121,7	2,22080292	0,81	115,3
53	126,8	2,39245283	0,81	75,8
53,3	128,3	2,407129456	0,81	94,8
82,5	198,5	2,406060606	0,83	136,2
56	126,2	2,253571429	0,79	96,4
56,1	121,6	2,167557932	0,76	115,8
58	140,6	2,424137931	0,88	173,2
57,1	149,5	2,61821366	0,87	114,6
64,4	127	1,972049689	0,66	104,7
55,9	139	2,486583184	0,85	74,4
61,8	139,8	2,262135922	0,82	84,7
56,8	126,2	2,221830986	0,8	86,5
53,4	107,9	2,020599251	0,73	60,5
56,7	118,1	2,082892416	0,74	95,9
56	149,9	2,676785714	0,95	67,6

67,8	155	2,286135693	0,79	58,2
56,4	174,8	3,09929078	1,117362581	94,9
57,7	127,1	2,202772964	0,743828983	46,3
59,6	146,8	2,463087248	0,803944649	56,4
54,6	142	2,600732601	0,89	69
62,6	154,1	2,461661342	0,82702267	73,2
55,5	114,3	2,059459459	0,703582893	83,5
56,4	152,8	2,709219858	0,833173068	58,1
63,7	173,3	2,720565149	0,872097539	76,4
63,5	177,3	2,792125984	0,966636753	97,7
63,2	153,9	2,435126582	0,84647479	82,9
59,1	136,5	2,30964467	0,79732574	74,9
50,8	130,2	2,562992126	0,868756556	77,3
108,2	210,4	1,944547135	0,677043931	75,1
56,5	153,4	2,715044248	0,953063627	95,9
53,6	131,8	2,458955224	0,810485536	69,2
53,7	141,7	2,638733706	0,897000851	116,4
63,1	149,3	2,366085578	0,804317909	106,2
51,6	116,4	2,255813953	0,756920706	128,1
54,6	151,8	2,78021978	0,868719783	80,6
57,5	145,8	2,535652174	0,792301106	80,95
67,3	146,3	2,17384844	0,750527205	71
59	141,8	2,403389831	0,829777007	79,9
